# Application of an Improved Correlation Method in Electrostatic Gait Recognition of Hemiparetic Patients

**DOI:** 10.3390/s19112529

**Published:** 2019-06-03

**Authors:** Shanshan Tian, Mengxuan Li, Yifei Wang, Xi Chen

**Affiliations:** State Key Laboratory of Mechatronics Engineering and Control, Beijing Institute of Technology, Beijing 100081, China; shanshanbit@126.com (S.T.); formlmx@126.com (M.L.); wangyifeibit@163.com (Y.W.)

**Keywords:** gait analysis, gait correction, electrostatic gait signal, improved Detrended Cross-Correlation Analysis cross-correlation coefficient

## Abstract

Hemiparesis is one of the common sequelae of neurological diseases such as strokes, which can significantly change the gait behavior of patients and restrict their activities in daily life. The results of gait characteristic analysis can provide a reference for disease diagnosis and rehabilitation; however, gait correlation as a gait characteristic is less utilized currently. In this study, a new non-contact electrostatic field sensing method was used to obtain the electrostatic gait signals of hemiplegic patients and healthy control subjects, and an improved Detrended Cross-Correlation Analysis cross-correlation coefficient method was proposed to analyze the obtained electrostatic gait signals. The results show that the improved method can better obtain the dynamic changes of the scaling index under the multi-scale structure, which makes up for the shortcomings of the traditional Detrended Cross-Correlation Analysis cross-correlation coefficient method when calculating the electrostatic gait signal of the same kind of subjects, such as random and incomplete similarity in the trend of the scaling index spectrum change. At the same time, it can effectively quantify the correlation of electrostatic gait signals in subjects. The proposed method has the potential to be a powerful tool for extracting the gait correlation features and identifying the electrostatic gait of hemiplegic patients.

## 1. Introduction

With the aging of the global population, strokes and other neurological diseases are occurring more frequently. Hemiparesis is one of the common sequelae of these diseases. Hemiplegic patients with body motor dysfunction often show a hemiplegic gait, among other symptoms. Assessing and restoring the walking ability of patients is the main objective of rehabilitation treatment for hemiplegic patients. The literature shows that the use of a variety of sensing methods to obtain hemiplegic gait information in clinical manifestations of hemiparesis [1,2], in order to study the kinematics and mechanics of gait signals and to objectively and quantitatively evaluate the pathological gait characteristics of hemiplegic patients, can provide effective support for the diagnosis and rehabilitation of hemiplegic patients [3,4].

At present, there are two main methods of gait measurement; one is the contact measurement method, which is usually obtained through the subject wearing an inertial unit sensor [5,6] or photoelectric sensor [7,8]; the other is the non-contact method, in which a video analysis system is used [9]. However, these common methods have some limitations. For example, wearing a sensor will affect the natural gait of the subject, while a video system compromises the privacy of the subject and is associated with data processing difficulties. Because of its non-contact, non-intrusive, and long-term continuous monitoring of gait signals, electrostatic detection has gradually become a research focus in the field of gait measurement [10,11]. In Chen’s work [12], the equivalent capacitance model of the human body was established by theoretical deduction, and the correctness of the model was verified by simulations and measured signals. In the paper by Li [13], the gait parameters of the “gold standard” plantar pressure system were compared with the electrostatic measurement system, and this proved that the gait time parameters can be effectively measured by the electrostatic field sensing method. The electrostatic gait signal is a non-stationary time series, which contains various motion parameters of the human body, including time, frequency and nonlinear parameters, among others. The related research on feature extraction of the gait signal not only helps explain gait signal fluctuations, obtaining more gait parameters, but also helps to analyze the physiological health status of the human body.

Correlation is an important feature of the non-stationary time series. Correlation analysis of the electrostatic gait signal can demonstrate the inherent characteristics of the signal, and the implicit correlation between the signals. Detrended fluctuation analysis (DFA), first proposed by Peng, is used to study the long-range fluctuation of DNA sequences [14]. After that, DFA was widely used in financial time series [15], meteorological time series [16], and physiological signal series [17,18]. However, the traditional DFA cannot describe a complex biological signal sequence accurately because of its single scale index. In order to better describe the correlation of non-stationary time series, the bi-index analysis method and local index derivation method are studied in the works by Peng and Castiglioni [19,20]; however, there is a limitation in that they cannot describe in detail the dynamic correlation of the signal. Xia Jianan et al. [21] proposed a local moving window combined multiscale detrended fluctuation analysis (MSDFA) method in order to study the dynamics of the correlation of signals, wherein the scale index spectrum is obtained by fitting *logF*(*n*) and *log*(*n*) in a local moving window to distinguish the ECG signals of healthy and pathological groups, and effectively avoid the influence of extreme values with this method. The correlation expansion of a non-stationary time series can obtain the cross-correlation between two non-stationary time series. The Detrended Cross-Correlation Analysis (DCCA) method proposed by Podobnik and Stanley [22] was first used to calculate the cross-correlation between two non-stationary time series. It has been widely used in financial [23,24], atmospheric [25], physiological, and other fields [26]. However, the single scaling index in the DCCA results still has similar inadequacies to DFA; that is, only a single parameter is used to describe the sequence characteristics. In order to solve this problem, Yin Yi et al. [27] applied the improved multiscale detrended cross-correlation analysis (MSDCCA) method in order to obtain the scaling index spectrum. This method can better show the dynamic changes of the scaling index in different scaling windows, and effectively avoid the influence of outliers, so it has good robustness. 

In order to quantitatively analyze the correlation of non-stationary signals, Zebende proposed a DCCA cross-correlation coefficient method based on DFA and DCCA [28]. However, we found that when this method was used to calculate electrostatic gait signals, the results would fluctuate abnormally near the crossover point [29,30]. Further, the traditional DCCA cross-correlation coefficient spectrum of electrostatic gait signals of the same type of subjects has random and incomplete similarity, so it was difficult to distinguish hemiplegic patients from healthy control subjects by the DCCA cross-correlation coefficient. In order to solve this problem, an improved DCCA cross-correlation coefficient method is proposed in this paper. Local moving windows are used instead of the fixed scales in the original algorithm. The results of the improved algorithm show a uniform and regular single peak structure at the crossover points, and have a stable curve structure; moreover, the results of different electrostatic gait signals for hemiplegic patients showed a relatively consistent trend. This method can effectively improve the stability of the DCCA cross-correlation coefficient, and quantify the correlation level of electrostatic gait signals under different scales.

The rest of this paper is arranged as follows: The second part will introduce the principle of electrostatic sensing method and the experimental process of electrostatic gait signal acquisition, as well as how to obtain electrostatic gait signals of hemiplegic patients and healthy control subjects; the third part will introduce in detail the improvement of the traditional DCCA cross-correlation coefficient method; and the fourth part uses the traditional method to analyze the electrostatic gait signals of subjects, from which we find that the traditional method has some shortcomings in the analysis of electrostatic gait signals. Then, we use the improved method to analyze the electrostatic gait signals. Through a comparison between the traditional method and the improved method, it can be concluded that the improved method is more suitable for the correlation analysis of electrostatic gait signals. This method can be better applied to the correlation analysis of human electrostatic gait signals. 

## 2. Methods

### 2.1. Principle of Electrostatic Field Induction in the Human Foot

**Figure 1:**
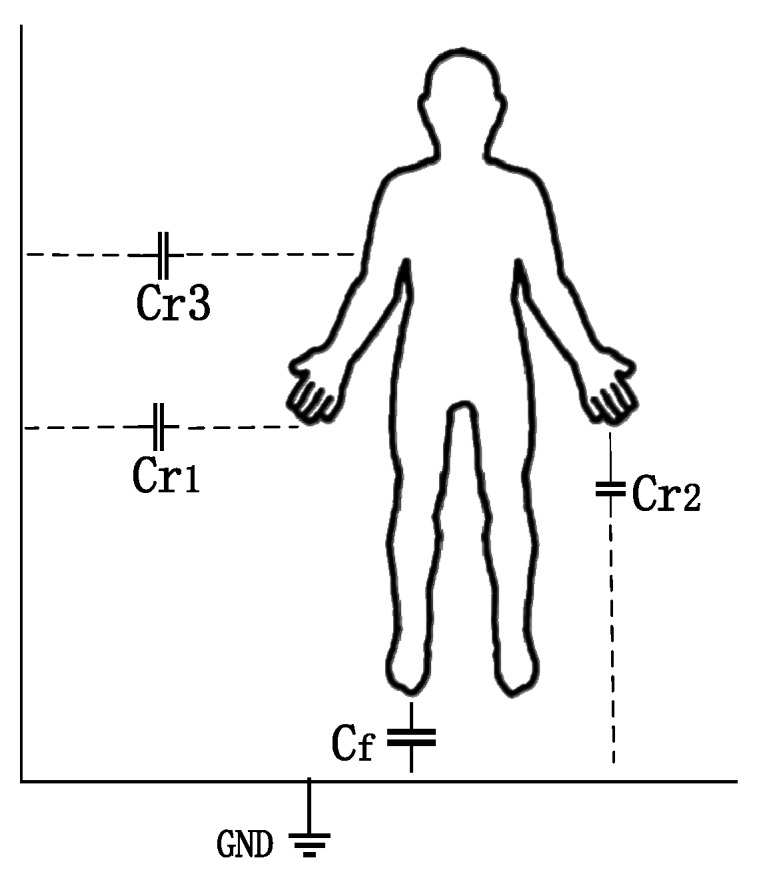
Sketch of the human body equivalent capacitance model.

The electrostatic phenomenon is a ubiquitous physical phenomenon. The human body is charged with electrostatic energy during movement due to friction between the body and the clothes, as well as friction between the sole and the ground [31,32]. Therefore, with the movement of the foot during walking, the electric field around the human body will change [33]. In Chen’s research [12], based on the principle of the electrostatic field, the equivalent model of the human body was established. Because the human body has a certain charge, it will produce equivalent capacitance with the surrounding environment, including the direct coupling capacitance *C_f_* generated by human feet contacting the ground through the sole, and other capacitance *C_r_* (*i* = 1, 2 ...). The two capacitors are connected in parallel to form the total capacitance of the human body (Formula (1)). The equivalent capacitance model of the human body is shown in Figure 1.

(1) $$C_{h}=C_{f}+\sum_{i=1}^{\infty }C_{ri}$$

The left and right feet alternately leave the ground when walking. The capacitance between the foot and the ground is equivalent to a variable capacitance which connected to the height of the foot from the ground in series with *C_f_*. The capacitance value becomes *C*, resulting in dynamic changes of electrostatic field around the human body. As in the literature [12], assuming that the charge of the human body is *Q_B_*, the induced current *I* placed on the sensing electrode at a certain distance from the subject is shown in Formula (2).

(2) $$I=\frac{dQ_{B}}{dt}\;=C\frac{dU_{B}}{dt}\propto \frac{1}{S_{s}}\frac{d}{dt}(h(t))-\frac{h(t)}{S_{s}{}^{2}}\frac{dS_{s}}{dt}$$

*U_B_* is the induced potential generated during human walking, *h*(*t*) is the height function of human feet leaving the ground when walking, and *S_s_* is the effective bottom area of *h*(*t*) from the ground height. The first item in Formula (2) represents the current generated by the foot movement before the foot leaves the ground, and the second item in Formula (2) represents the current caused by the foot and leg movement after the foot leaves the ground completely. Therefore, when the tester moves near the electrodes, we can obtain the electrostatic induction current caused by human motion under non-contact conditions, and then obtain the relevant parameters of the gait by analyzing the current waveform obtained.

### 2.2. Human Electrostatic Gait Measurement System

**Figure 2:**
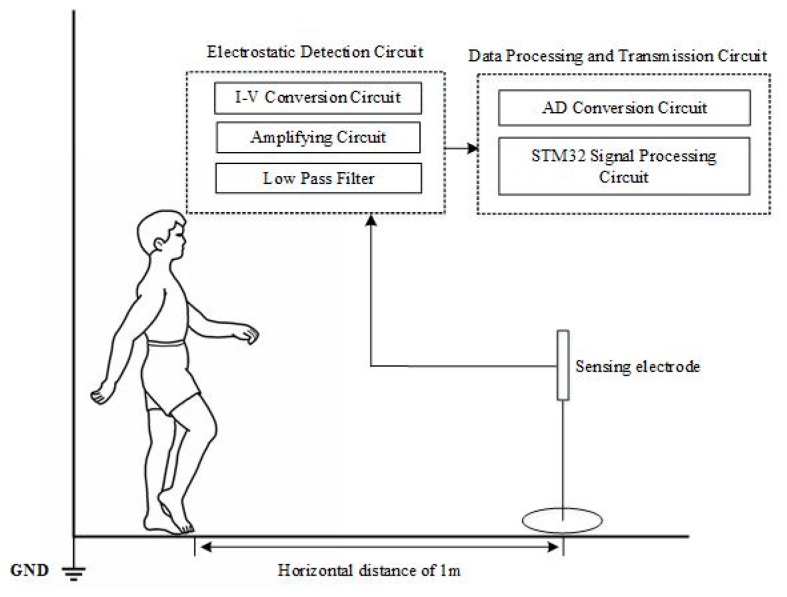
Schematic of the electrostatic sensing system.

The electrostatic signal sensing system includes the induction electrode, electrostatic sensing circuit, signal acquisition and processing circuit. The system schematic diagram is shown in Figure 2. In this system, a copper foil planar electrode is used as the electrode material. The shape of the electrode is a circle with a diameter of 90 mm. The electrostatic sensing circuit allows the amplification and filtering of the electrostatic signal, and realizes the conversion and amplification from the induced charge to the current to the voltage, thereby converting the weak induced charge amount into a voltage signal that can be processed. A low-pass filter with a cut-off frequency of 20 Hz was added before A/D conversion in order to prevent the measured signal from being disturbed by the power frequency signal of the power grid. The signal acquisition and processing circuit converts the amplified and filtered analog signals into digital signals using an A/D conversion with high precision and a wide voltage input, and then processes the collected signals using a microcontroller unit. The sampling frequency of the system is 1 kHz, and the sampled data is sent to a personal computer for storage and processing. According to [13], the current intensity of the induced current is inversely proportional to the distance between the human body and the induction electrode, and the maximum induced current can be generated when the human body directly faces the induction electrode. Therefore, in order to obtain a better gait electrostatic signal, the subject is required to tread 1 m in front of the electrostatic induction electrode.

### 2.3. Gait Measurement Experiment

In this experiment, 10 hemiparetic patients and 10 healthy control subjects participated in data collection. The experiment recruited 10 hemiparetic patients (six males and four females) from the Zhongshan People’s Hospital of Guangdong Province, China, as the pathological group. Their average height was 1.68 m (height range: 1.58~1.76 m), the average weight was 68.7 kg (56~78 kg), and the average age was 46 years (age range: 31~60 years old). The selection criteria for the hemiparetic patients was: (1) Unilateral hemiparesis after their first stroke, and the condition has been confirmed by computed tomography (CT) or nuclear magnetic resonance (MRI) imaging; (2) able to continuously step or walk for at least three minutes without any help or auxiliary equipment; (3) understands the external orders and follows the experimental procedures; (4) suffers no other diseases known to affect the gait [34]. This research was approved by the Ethics Committee of Zhongshan People’s Hospital, and an informed consent form was signed by each subject.

The healthy control group had no neurological damage (six males and four females). Their average height was 1.73 m (height range: 1.58~1.81 m) and the average weight was 61.7 kg (45~78 kg). The average age was 28 years (age range: 24–33 years old).

All subjects gave their informed consent for inclusion before they participated in the study. The study was conducted in accordance with the Declaration of Helsinki, and the protocol was approved by the Ethics Committee of Zhongshan People’s Hospital.

In the experiment, the electrostatic testing equipment was placed on a triangle stand at a height of 1 m from the ground. The healthy control group and the hemiplegic patients wore ordinary rubber sole shoes, and they were required to tread at a distance of 1 m in front of the electrostatic induction electrode. The subjects were required to tread in situ at a normal pace. Two groups of signals from the hemiplegic patients and healthy controls were obtained; that is, a total of 40 groups of electrostatic gait signals were collected, each of which was about 60 s in length. The ambient temperature and humidity ranged from 20 to 25 °C, and from 50% to 60%, respectively.

### 2.4. Preprocessing of the Experimental Data

**Figure 3:**
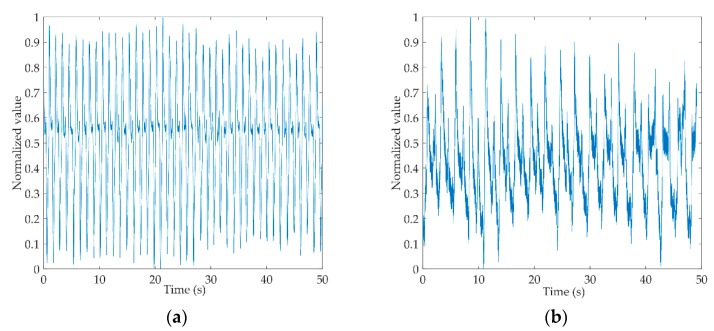
Schematic diagram of the original electrostatic gait signal. (**a**) Preprocessed electrostatic gait signals of a healthy control; (**b**) Preprocessed electrostatic gait signals of a hemiplegic patient.

Figure 3a,b shows the original gait signals of a healthy control person and a hemiplegic patient. The signals are digitally filtered and normalized to intercept 50 s of data from the complete signals. From the figure, we can see that the amplitude of the healthy control is more stable than that of the hemiplegic patient, and the gait cycle of the healthy control is shorter than that of the hemiplegic patient.

### 2.5. Improved DCCA Cross-Correlation Coefficient Method

The DCCA cross-correlation coefficient method has the characteristic of quantifying the cross-correlation level between two non-stationary time series. The DCCA cross-correlation coefficients of two non-stationary time series *x*(*i*) and *y*(*i*) are defined as:

(3) $$\rho_{DCCA}\left(n\right)=\frac{f_{DCCA}^{2}\left(n\right)}{f_{DFAx}\left(n\right)\cdot f_{DFAy}\left(n\right)}$$

The DCCA cross-correlation coefficient is calculated from the DFA and DCCA of the sequence. When dealing with electrostatic gait signals, this method is sensitive to the fluctuations of *f_DFA_* and *f_DCCA_*; it cannot accurately and quantitatively reflect the correlation level. The local moving window can display the details of physiological signal dynamic change and has good robustness. This paper proposes an improved DCCA cross-correlation coefficient algorithm based on the local moving window.

The improved DCCA cross-correlation coefficient method is described as follows: calculate the sample sequence to get the detrended variance function *f_DFAx_*(*n*) and *f_DFAy_*(*n*), and detrended covariance function *f_DCCA_*(*n*), where *n* is the scale. The fluctuation function curves are divided by the local moving window, then the data in each window is fitted. The expression of the fitted line can be expressed as (*α*·*n* + *b*), where *α* is the slope of the line, which can also be regarded as the scale index in each local window, while *b* is the only parameter of the currently fitted straight line in the correlation analysis [21,27]. We are only focused on using the scale index to quantify the correlation, so parameter *b* is not discussed. Therefore, every window DCCA cross-correlation coefficient can be represented as:

(4) $$\rho_{DCCA}\left(n\right)=\frac{{\left(\alpha_{DCCA}\cdot n\right)}^{2}}{\left(\alpha_{DFAx}\cdot n\right)\cdot \left(\alpha_{DFAy}\cdot n\right)},\left(n\in wi=\left[c,d\right],i=1,2,\ldots ,m\right)$$

The method for dividing the local moving window is described as follows: For the sample data with the data length *N*, set the range of *n* as (5 < *n* < *N*/10), then the partial moving window is divided. *w_i_* = [*c*, *d*] (*i* = 1, 2, …, *m*) is expressed as each local moving window, *c* is the starting value, *d* is the ending value, the starting value interval is 5, and the ending value is five times the starting value, *m* is the number of local moving window. *s_wi_* indicates the scaled median value of each window, *α_wi_* indicates the scale index in the corresponding window, and *α_w_* = {*α*_*w*1_, *α*_*w*2_, …, *α_wm_*} is the scale index spectrum.

After calculating all the window data, the above formula can be improved to Formula (5):

(5) $$\rho_{MSDCCA}\left(s_{wi}\right)=\frac{{\left(\alpha_{wi,DCCA}\right)}^{2}}{\left(\alpha_{wi,DFA}\right)\cdot \left(\alpha_{wi,DFA}\right)},\left(i=1,2,\ldots ,m\right)$$

We call *ρ_MSDCCA_*the multi-scale detrended cross-correlation coefficient. In the results, *ρ_MSDCCA_* > 0 indicates that there is positive cross-correlation between sequences, while *ρ_MSDCCA_* < 0 indicates that there is an anti-cross-correlation between sequences. The dynamics of the sequence and the inherent complex structure of the sequence can be observed from the curve of *ρ_MSDCCA_* changing with *s_wi_*.

## 3. Results

In this part, we analyzed the electrostatic gait signal of the hemiplegic patients and the healthy control subjects with the traditional method. We found that the traditional method had some deficiencies in the analysis of the electrostatic gait signal correlation. Then, the electrostatic gait signal was analyzed by the improved method proposed in this paper. The results showed that the improved method improved the stability and accuracy of the data results compared with the traditional method, and helped to better identify the electrostatic gait signal of hemiparesis.

### 3.1. Analysis of the Electrostatic Gait Signal with the Traditional Method

**Figure 4:**
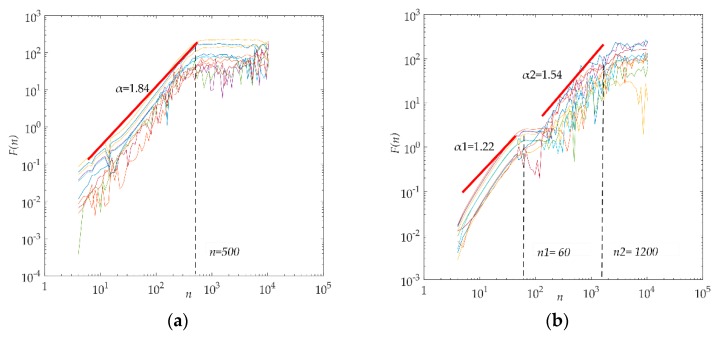
DCCA analysis of healthy controls (**a**) and hemiplegia patients (**b**).

DCCA is used to analyze the correlation between two non-stationary time series, and has been widely used in finance, atmosphere, physiology, and other fields [23,24,25,26]. In this paper, the electrostatic gait signals of hemiplegic patients and healthy control subjects are analyzed with the DCCA method, and the logarithmic curve of the cross-correlation fluctuation function and the scale of the electrostatic gait signals are obtained, as shown in Figure 4. From graph (a), we can see that all the cross-correlation wave functions of healthy controls have good consistency, and the curves have a crossover point (*n* = 500). The cross-correlation index spectrum of the electrostatic gait signals of the healthy controls is obviously divided into two sections, with a single index (*α_DCCA_* = 1.84). From graph (b), we can see that the cross-correlation wave function curves of all hemiplegic patients are scattered. There are two crossover points (*n*_1_ = 60, *n*_2_ = 1200). The cross-correlation index spectrum of the electrostatic gait signals of hemiplegic patients is obviously divided into three segments. There are two indices for the electrostatic gait signals of hemiplegic patients (*α*_*DCCA*1_ = 1.22, *α*_*DCCA*2_ = 1.54). In Figure 4b, the waveform functions of different samples of hemiplegic patients show chaotic waveform characteristics after the first crossover point. This phenomenon is related to the difference of walking ability between hemiplegic patients, but the waveform characteristics cannot be explained by a single index.

Although the cross-correlation wave function spectrogram of the electrostatic gait signals of subjects calculated by the DCCA method can show the difference between them, the long-range cross-correlation of the electrostatic gait signals of hemiplegic patients can be seen from a single index that is weaker than that of the healthy controls. However, this difference is small, and the cross-correlation index cannot quantify the strength of cross-correlation between signals.

**Figure 5:**
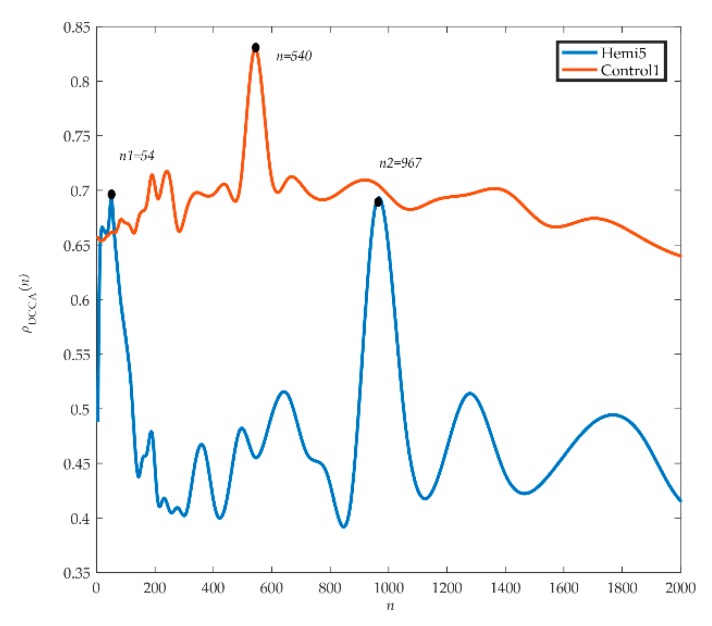
Spectrum of *ρ_DCCA_* in healthy control 1 and hemiplegic patient 5.

DCCA cannot quantify the correlation of electrostatic gait signals. In this paper, the DCCA cross-correlation coefficient method is used to analyze the electrostatic gait signals quantitatively. This method is also widely used in physiological signals [35]. The DCCA cross-correlation coefficient method is defined by DFA and DCCA to quantitatively study the cross-correlation between non-stationary time series. The electrostatic gait signals of hemiplegic patients and healthy controls were calculated and analyzed by the DCCA cross-correlation coefficient method. Figure 5 shows the analyzed electrostatic gait signals of hemiplegic patient 5 (hemi 5) and healthy control 1 (control 1). We can see from the graph that the spectrum of *ρ_DCCA_* in hemiplegic patient 5 fluctuates greatly. The first peak (*ρ_DCCA_* = 0.69) appears at the scale *n*_1_ = 54, and then the curve shows a downward trend and a disorderly fluctuation trend. The second peak (*ρ_DCCA_* = 0.68) appears at the scale *n*_2_ = 967, and then the curve decreases and produces two small peaks. The scales corresponding to wave peaks are similar to those of crossover points in DCCA, and on all scales, *ρ_DCCA_* = 0.54 ± 0.06. For healthy control 1, the fluctuation of the spectrogram of *ρ_DCCA_* was relatively stable, but there was a peak fluctuation on scale *n* = 540 (*ρ_DCCA_* = 0.83) and *ρ_DCCA_* = 0.69 ± 0.06 on all scales.

The DCCA cross-correlation coefficient can quantify the correlation of the electrostatic gait signals in hemiplegic patients and healthy controls. After DCCA cross-correlation coefficient analysis of data from all hemiplegic patients and healthy controls, we found that the fluctuation of *ρ_DCCA_* near the crossover point was a common phenomenon, showing a chaotic fluctuation of one main peak and several sub-peaks. The trend of the DCCA cross-correlation coefficient spectrum of electrostatic gait signals of the same type of test population was random and similar.

Due to the different fluctuation characteristics of time series on different time scales, the crossover point phenomenon appears in the DFA and DCCA results, and the complex fluctuation phenomenon appears near the crossover point of the cross-correlation index spectrum, which will affect the quantitative judgment of the cross-correlation. The traditional method has some limitations in the analysis of electrostatic gait signals, so we need to improve the traditional method for better analysis of electrostatic gait signals.

### 3.2. Improved DCCA Cross-Correlation Coefficient Method for Electrostatic Gait Signal Analysis

**Figure 6:**
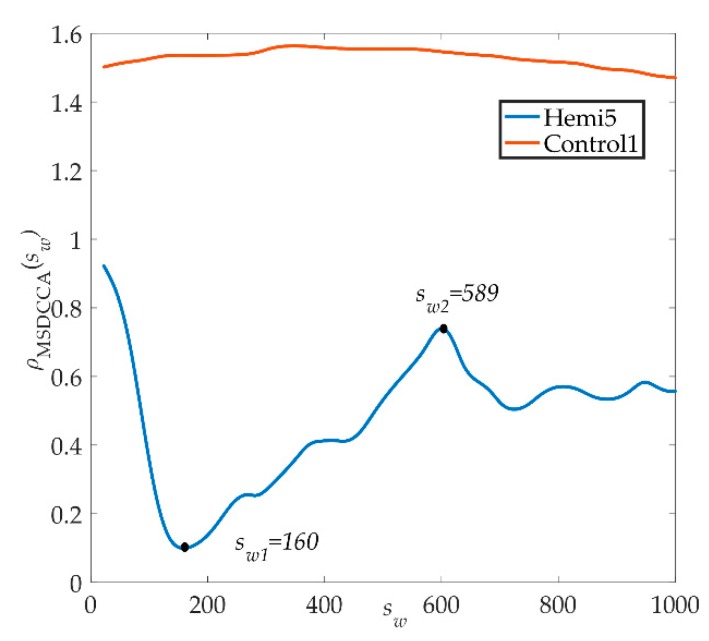
*ρ_MSDCCA_* spectrograms of healthy control 1 and hemiplegic patient 5.

Because the traditional DCCA cross-correlation coefficient method has some limitations, the improved method is used to analyze hemiplegic patient 5 and healthy control 1. As shown in Figure 6, we can see from the graph that the spectrum of *ρ_MSDCCA_* in hemiplegic patient 5 first decreases, then rises, and finally tends to be stable. The scale window *s*_*w*1_ = 160 has a trough, the correlation coefficient spectrum in the 160 < *s_w_* < 589 interval shows an increasing trend, the scale window *s*_*w*2_ = 589 has a peak, and the correlation coefficient of *s_w_* > 589 first decreases to a stable trend. The correlation coefficients near the peak and trough did not fluctuate significantly in the results of *ρ_MSDCCA_*. In contrast, the spectral line of healthy control 1 was relatively stable and showed no obvious fluctuation in the whole scale window, and the range of healthy control 1 was *ρ_MSDCCA_* = [1.4, 1.6].

**Figure 7:**
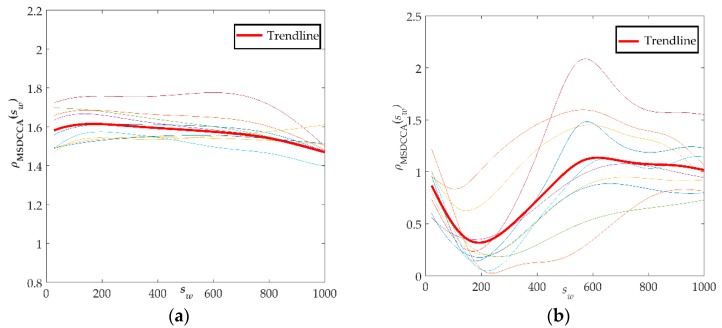
Result curve of *ρ_MSDCCA_*. (**a**) Analytical curves of *ρ_MSDCCA_* of all healthy controls with respect to the change of moving windows; (**b**) Analytical curves of *ρ_MSDCCA_* with the moving window in all hemiplegic patients.

Finally, the improved method was used to analyze the electrostatic gait signals of all subjects, and the correlation coefficient spectra of *ρ_MSDCCA_* of hemiplegic patients and healthy controls were obtained, as shown in Figure 7. The red bold line in the figure represents the average of all data changes. Figure 7a is a spectrogram of the coefficients of *ρ_MSDCCA_* of the healthy control group. The curve tends to be stable in the window scale range. The range of *ρ_MSDCCA_* is [1.3, 1.8], and the mean and standard deviation are 1.45 ± 0.12. The gait signals of the healthy control group have a better correlation. The coefficient curve of *ρ_MSDCCA_* in the hemiplegic patient group is shown in Figure 7b. From the graph, we can see that the curve first has a downward trend and then an upward trend, and tends to be stable. The fluctuation of the *ρ_MSDCCA_* curve in the hemiplegic patients is closely related to the crossing point. The stability range of *ρ_MSDCCA_* is [0.6, 1.6], and the mean and standard deviation are 0.93 ± 0.26.

From the spectrum of hemiplegic patients and healthy controls calculated by the improved method, we can clearly see the difference between them, meaning it is possible to effectively distinguish the hemiplegic patient from the healthy control easily. In addition, it can be seen from the spectrum of *ρ_MSDCCA_* that the local variation trend of correlation coefficients near the crossover point is identical, with many similarities and good stability. Moreover, the improved method can quantitatively show the difference between the correlation of the gait signals of two groups of subjects, and it can also reflect the differences of walking ability between individual hemiplegic patients.

## 4. Conclusions

This study analyzes the correlation between the electrostatic gait signals of hemiparetic patients and healthy controls by using an improved method which combines the local moving window method and traditional DCCA cross-correlation coefficient method. Using the improved method to analyze the electrostatic gait signal can mitigate the weaknesses of the traditional method, such as the random trend changes and incomplete similarity. In this paper, the electrostatic gait signals of hemiparetic patients and healthy controls were collected, and the improved method was used for gait analysis. Compared with the *ρ_MSDCCA_* coefficient of the healthy control group, the mean value of the *ρ_MSDCCA_* coefficient of the hemiparetic patients was smaller, and the trend of change was more complicated, reflecting that the gait stability of the hemiparetic patients was weaker than that of the healthy controls, additionally, a poor correlation was obtained. The improved method can obtain the dynamic changes of the scale index under the multi-scale structure, and quantify the gait difference between the hemiparetic patients and the healthy controls.

We only collected one minute of the patients’ gait signal data due to their weakened mobility. This paper does not consider the difference in the gait signal between individual patients due to different rehabilitation stages, which can be considered a limitation. In future research, we will obtain more abundant experimental data, and conduct more in-depth research based on the different rehabilitation stages of hemiparetic patients, and then obtain more accurate and effective characteristic parameters. In the future, multiple characteristic parameters could be used to analyze various abnormal gaits, such as the hemiparesis and Parkinson gaits.
